# Editorial: Abiotic stress alleviation in plants: morpho-physiological and molecular aspects

**DOI:** 10.3389/fpls.2023.1295638

**Published:** 2023-10-03

**Authors:** Mahmoud Magdy, Mohammad Golam Mostofa, Mehdi Rahimi, Diaa Abd El Moneim

**Affiliations:** ^1^ Genetics Department, Faculty of Agriculture, Ain Shams University, Cairo, Egypt; ^2^ Plant Biology Department, Faculty of Biology, Murcia University, Murcia, Spain; ^3^ Department of Energy Plant Research Laboratory, Michigan State University, East Lansing, MI, United States; ^4^ Department of Biochemistry and Molecular Biology, Michigan State University, East Lansing, MI, United States; ^5^ Department of Biotechnology, Institute of Science and High Technology and Environmental Sciences, Graduate University of Advanced Technology, Kerman, Iran; ^6^ Department of Plant Production (Genetic Branch), Faculty of Environmental Agricultural Sciences, Arish University, El-Arish, Egypt

**Keywords:** plant resilience, abiotic stressors, molecular strategies, physiological adaptation, global agriculture, environmental challenges, ecosystem sentinels, thematic research collection

## Research topic overview

Plants, our ecosystem’s guardians, endure amidst ever-changing environmental challenges. Abiotic stresses, including drought and temperature extremes, continually test their resilience. Unraveling the mechanisms of abiotic stress tolerance in plants through molecular and physiological lenses is central to contemporary plant research. Within this scientific realm, understanding abiotic stress response is crucial. Each article in this Research Topic offers insights, sketching the diverse tactics plants deploy for survival. These pieces highlight the molecular pathways and physiological shifts plants use to counter abiotic adversities. In this editorial, we traverse a vibrant mosaic of research, organizing articles into themes highlighting plants’ varied coping mechanisms. Beyond these insights awaits a realm of potential. The repercussions of our findings reach beyond academic realms, influencing global agriculture, food availability, and environmental balance. As we navigate the intricate labyrinth of abiotic stress adaptation, the featured articles shed light on the multifaceted dimensions of plant reactions. While particular articles seamlessly align with our designated sub-thematic clusters, others straddle multiple themes, underscoring the complexity of this research paradigm. Our editorial consortium has endeavored to contextualize each article based on the work’s primary focus within the most apt sub-theme, as delineated below:

## Sub-theme 1: investigating physiological, biochemical, and metabolic responses to abiotic stressors: implications for plant adaptive mechanisms

In the realm of plant science, understanding plants’ responses to abiotic stressors like drought and heat is crucial. As these challenges threaten food security, research focuses on plants’ multifaceted survival tactics. The ensuing studies illuminate a range of physiological, genetic, and agronomic strategies, revealing intricate metabolic pathways that equip plants to navigate these adversities.

A group of the published articles predominantly focused on plants’ visible and immediate response to stress. In the context of drought amelioration strategies, Mahmud et al. elucidated an innovative tactic. They discovered that after acetic acid priming and subsequent exposure to drought stress induced by PEG, maize seeds manifested remarkable resilience. Such seeds displayed enhanced growth parameters, predominantly in shoot and root elongation. Concurrently, there was a notable augmentation in the plant’s inherent water conservation strategies and photosynthetic efficiency, underscoring acetic acid’s potential to fortify drought resilience via molecular and physiological avenues. The indispensable role of proteins in abiotic stress defense mechanisms was highlighted by (Yan et al.). They emphasized the significance of the OsOLP1 osmotin-like protein in rice, which, upon overexpression, conferred remarkable drought resistance. The treated plants displayed enhanced stomatal regulation, impressive leaf water conservation, and superior survivability, fundamentally supported by an escalation in lignin and proline concentrations. This insinuates that the strategic management of such proteins could be paramount in enhancing crop resilience. Ding et al. probed the thermotolerance mechanisms in rice by evaluating the hst1 mutant, steered by the DST transcription factor. A pronounced thermotolerance was discerned, attributed to elevated ROS concentrations, which stimulated heat shock protein expression. Such proteins are pivotal in cellular homeostasis during thermal stress, accentuating the DST transcription factor’s potential in fortifying plant resistance amidst diverse abiotic challenges. Wang et al. underscored the potential of H_2_O_2_ priming to mitigate the detrimental impacts of waterlogging in maize, revealing enhancements in photosynthetic efficiency and overall plant vitality, suggesting applicative prospects in inundated terrains.

Under the same sub-theme, additional works dived into several plants’ underlying genetic and molecular strategies. Gene expression plays a quintessential role in abiotic stress resistance. Shao et al. explored the CdWRKY2 gene in bermudagrass, a species distinguished for its innate stress resilience. There was a conspicuous association between the CdWRKY2 gene expression and its susceptibility to salt and ABA. A nuanced interplay in lateral root responses, striking a balance between ABA sensitivity and auxin accumulation, was associated with this gene, hinting at the prospects of genetic engineering for augmented salinity tolerance in agronomic crops. Zhang et al. probed into the molecular dynamics of plant responses, unveiling the critical role of H+-pyrophosphatases during nitrogen-deficient conditions. The study spotlighted the relevance of AtAVP1 in Arabidopsis and EdVP1 in *Elymus dahuricus*, exposing a potential regulatory circuit influencing plant acclimatization to nitrogen-scarce settings, thus suggesting possible avenues for enhancing nitrogen assimilation in crops. Li et al. delved into the molecular adaptability of sugar beet (*Beta vulgaris* L.) under nitrogen paucity. Employing transcriptome profiling, they discerned a meticulous balance between energy preservation and oxidative damage defense, suggesting adaptive strategies for other crops under nitrogen constraints. Bai et al. delineated the intricate regulatory dynamics of calcium ion signaling and its receptors in plants, elucidating the multifaceted interactions of these entities in orchestrating plant responses to diverse stressors and suggesting pathways for optimizing plant resilience in adversarial environments.

More works have emphasized agronomic and adaptive practices and techniques to improve plant stress resistance. Wu et al. accentuated the merits of grafting by juxtaposing grafted and non-grafted tomato variants under saline conditions. The grafted variants manifested superior salinity resilience and a more harmonized gene expression trajectory, signifying the potentiality of grafting as a modality to confer salt resistance and decipher key metabolic conduits and transcriptional regulators. Karimian et al. illuminated the efficacy of nano fertilizers in bolstering drought resistance in *Dracocephalum kotschyi*. Their findings suggested that zinc nanoparticle amendments could maintain resilience by modulating pivotal biochemical markers, hinting at a promising direction for drought amelioration in diverse plant species. Miao et al. explored the intricate nexus between light quality and saline stress, proposing a strategy to fine-tune light spectra to alleviate salt stress in cucumber plants. Their empirical findings intimate that modulation of red to far-red light ratios can potentially mitigate the adverse repercussions of salt stress, outlining an avant-garde tactic for enhancing salinity resistance.

The array of studies showcased reveals the multifaceted strategies underpinning plant resilience. Researchers are amplifying our arsenal to strengthen crops amidst a shifting climate by intertwining physiological, molecular, and agronomic approaches. This fusion of methodologies could be pivotal for safeguarding food security and ecological harmony in the future.

## Sub-theme 2: molecular responses of stress-related genes and pathways for plant growth regulation and development under abiotic stress conditions

Molecular biology’s frontier vibrantly probes the genetic orchestration behind plant resilience amidst growing abiotic challenges. Grasping these genetic pathways is vital for boosting crop robustness and understanding ecological evolution in shifting climates. The highlighted studies unveil specific stress-linked genes and the overarching molecular tactics guiding plant evolution under diverse stress conditions.

Several articles in on this topic presented works that concentrate on a particular gene and its influence on abiotic stress resistance. Huang et al. delved deep into the genomic landscapes of the cereal *Setaria italica L.*, highlighting the significance of the SiNCED1 gene. Markedly upregulated under a gamut of abiotic stressors, including abscisic acid (ABA), osmotic perturbations, and saline conditions, the SiNCED1 gene, when overexpressed, induced a plethora of adaptive responses. These ranged from augmented drought resistance, orchestrated by elevated ABA concentrations and strategic stomatal closure, to influencing a complex network of ABA-responsive stress genes. Transitioning from cereal to medicinal trees, Zhang et al. provided a comprehensive genomic analysis of *Cyclocarya paliurus*. Their investigation unraveled a constellation of 159 basic helix-loop-helix (bHLH) transcription factors, pivotal regulators in plant stress responses. Systematic classification delineated these genes into 26 nuanced subfamilies, and subsequent functional assays under varied salt gradients spotlighted 12 genes demonstrating heightened responsiveness. CpbHLH36/68/146 emerged as paramount nodes in the salt tolerance regulatory network. Tang et al. spotlighted the HD-Zip transcription factor. Their inquiry into the *JcHDZ21* gene, a constituent of the HD-Zip I gene family in physic nut, revealed intriguing genetic dynamics. Transgenic plants showcasing JcHDZ21 overexpression presented heightened sensitivity to saline conditions, offering pivotal insights for potential breeding programs targeting stress-tolerant variants. Concluding this molecular journey, Jiao et al. focused on the salt-resistant halophyte *Limonium bicolor*. The epicenter of their research was the Lb1G04794 gene, intricately associated with salt gland development and resistance. Overexpression studies emphasized accelerated salt gland maturation in *L. bicolor* and elicited an array of beneficial adaptive responses in *Arabidopsis*, underpinning the gene’s paramount role in breeding salt-resilient cultivars.

Within the sub-theme frame, two additional studies focused on broader mechanisms or adaptations plants utilize under stress conditions. Cobo-Simón et al. ventured into the evergreen realm of *Cedrus atlantica*. Through a strategic analysis of seedlings subjected to diverse drought scenarios, their study unearthed potential loci of local adaptations. Astoundingly, the plant’s genomic responses showcased a spectrum ranging from immediate genomic recalibrations to more protracted alterations, complemented by post-drought recovery strategies. Their magnum opus discovery spotlighted specific genomic variances, mainly single nucleotide polymorphisms (SNPs) associated with transposable elements, earmarking drought-resilient phenotypes. Zeroing in on the metal-responsive gene pathways, Li et al. illuminated the genomic strategies of *Sedum plumbizincicola* under cadmium (Cd) stress. Central to their findings was the cadmium tolerance protein SpCTP3. Remarkably, genetic overexpression of SpCTP3 heralded an enhanced phytoremediation potential, elevating cadmium accumulation in the plant’s aerial and root architecture compared to wild types. These genetically manipulated plants also exhibited altered cadmium intracellular distribution paradigms and augmented antioxidative enzyme activities under Cd-induced stress.

Molecular research reveals the multifaceted intricacies of plant responses to abiotic adversities. From gene-focused analyses to comprehensive, adaptive strategies, nature’s resilience emerges from a rich interplay of genetic, cellular, and physiological narratives. This depth of understanding highlights an essential reality: decoding the molecular conversations within plants paves the way for a resilient and sustainable agricultural future.

## Sub-theme 3: comprehensive omics-based analyses to dissect abiotic stress coping mechanisms in plants

With the rapid advancements in omics technologies, researchers increasingly focus on an integrated understanding of plant responses at molecular, proteomic, transcriptomic, and metabolomic levels to dissect their adaptive mechanisms under various abiotic stresses. This multifaceted approach aids in elucidating the underlying mechanisms, revealing potential biomarkers, and suggesting innovative breeding and biotechnological strategies. Here, we cluster the research studies under this sub-theme based on their primary focus.

Under the effect of heavy metals on plants, the comprehensive analysis that can be conducted through the emerging omics approaches to understand the molecular mechanisms plants employ to tolerate such stresses is paramount. ShangGuan et al. elucidated the role of Germin-like proteins (GLPs) in *Oryza sativa* subjected to heavy metal stresses from Copper (Cu) and Cadmium (Cd). Utilizing hydroponic systems, their findings highlighted that GLP gene knockouts amplified the toxicological effects of Cd and Cu. Such mutants exhibited enhanced metal accumulation relative to wild-type (WT) plants. Intriguingly, altered sequestration patterns of Cu and Cd in the cell walls of these mutants were observed. Enhanced expression of the OsGLP8-2 gene bolstered lignin biosynthesis, crucial for maintaining cell wall structure, and concurrently upregulated antioxidative defense mechanisms against metalliferous stress.

In their evolutionary journey, plants have developed intricate physiological and developmental processes to cope with varying environmental stresses. Peng et al. delineated the pivotal role of Actin depolymerization factors (ADFs) in plant health and adaptative stress responses. Their investigations revealed that the CARK3 protein, via interaction with ADF4, modulates hypocotyl morphogenesis and functions as an intermediary in actin filament dynamics vis-à-vis drought stress. Distinct phenotypic alterations were evident in plants with targeted genetic perturbations in these proteins. Liu et al. investigated the implications of homeodomain-leucine zipper proteins, emphasizing HAT5 derived from *Pyrus sinkiangensis*, across diverse stress scenarios. Their empirical evidence indicated that the heterologous expression of this gene in *Solanum lycopersicum* conferred augmented tolerance against drought and salinity but, conversely, heightened susceptibility to cold stress.

When subjected to environmental stresses, metabolism shifts and growth regulation adaptations in plants provide unique insights into their survival strategies. Zhang et al. executed a holistic metabolomic examination of *Zea mays*, probing the merits of hydropriming in augmenting its salt stress resistance. Their findings underscored specific metabolic pathways and growth mediators that become predominant during stress, revealing potential strategies for fortifying maize’s stress resilience. Zhao et al. embarked on an in-depth analysis of the adaptive responses of *Lotus corniculatus* under phosphorus-deficient settings. They demarcated crucial molecular pathways and genetic determinants pivotal for this adaptation, offering prospects for breeding strategies focused on optimizing phosphorus utilization.

Delving deep into the genomic blueprints of plants, researchers can elucidate the genetic orchestration and subsequent adaptive mechanisms under diverse abiotic stress conditions. Owusu et al. examined *Gossypium* spp.’s genomic dynamics under extended hypoxic waterlogging-induced conditions. Their combined transcriptomic and metabolomic evaluations pinpointed pivotal genetic elements and metabolic cascades operative under sustained hypoxia, suggesting potential molecular targets to enhance Gossypium’s adaptability against such adversities. Pakzad et al. unraveled the adaptive proteomic and metabolic pathways in *Pistacia vera* when subjected to osmotic stress due to salinity or drought. Their findings emphasized essential proteins and molecular cascades imperative for osmotic stress adaptation, suggesting innovative approaches for improving osmotic stress resilience in this species. Mikołajczak et al. presented an intricate analysis of *Hordeum vulgare*’s molecular responses, focusing on its flag leaf, under simultaneous drought and thermal stresses. Their analytical insights revealed the intricate molecular strategies this species employs to navigate co-occurring environmental hardships, which are valuable for subsequent breeding efforts to enhance *Hordeum vulgare*’s resilience.

The accumulated evidence from these diverse studies underscores the immense potential of leveraging omics-based approaches to gain a holistic understanding of plant responses under abiotic stresses. Such insights enrich our fundamental knowledge and pave the way for innovative approaches to enhance plant resilience in the face of mounting environmental challenges.

## Sub-theme 4: biotechnological interventions of abiotic stress tolerance mechanisms for improvement of crop stress resistance to abiotic stresses

Enhancing crop resilience against abiotic stresses is crucial in the ever-evolving realm of agricultural science. Biotechnological tools provide a window to refine plant adaptability. This sub-theme bridges advanced biotechnological methods with plant responses, revealing molecular intricacies, evolutionary strategies, and symbiotic affiliations. Together, these studies highlight biotechnology’s role in fortifying crops against environmental challenges, steering a path for sustainable agriculture in a dynamic global landscape.

The intricacies of molecular and genetic dynamics offer profound insights into plant adaptability amidst adverse environmental conditions, presenting an avenue for the augmentation of stress resilience through precise biotechnological interventions. In an analytical endeavor focusing on *Solanum lycopersicum*, a crop of global agronomic significance, Perveen et al. emphasized the implications of biotechnological tools to bolster drought resilience. Their methodological approach, incorporating advanced in silico models and contemporary sequencing paradigms, delineated the cardinal role of WRKY gene families, which function as quintessential transcriptional regulators in psychophysiological processes and stress-mediated responses. A notable discovery encompassed the molecular interaction dynamics between the phytohormone abscisic acid, synthesized in stress-induced scenarios, and WRKY protein structures, suggesting avenues for enhancing drought adaptability via biotechnological interventions. Delving into the evolutionary intricacies of plant adaptive mechanisms, Bawa et al. characterized the operational dynamics of the auxin efflux carrier, PINFORMED1 (PIN1), within the cellular plasmalemma. Their investigative focus revolved around the modulatory impact of PIN1 on pavement cells (PCs) and guard cells (GCs) in response to abiotic stressors such as desiccation and salinity. Their observations elucidated the differential morphogenesis of PCs and GCs predicated on distinct environmental challenges, accentuating the pivotal role of PIN1 in orchestrating epidermal cell development and proffering potential biotechnological interventions for plant resilience in hostile ecological milieus. The empirical exploration by Méndez-Cea et al. delineated the genetic landscape governing drought resilience in endogenous fir species, specifically *Abies pinsapo* and *Abies marocana*. Leveraging the ddRAD-seq genotypic platform, they discerned the underlying genetic architecture and identified loci potentially under environmental selective pressures. Identifying proteins intrinsic to abiotic stress responses, combined with genotype-environment association analyses, highlighted the potential vulnerabilities of these arboreal species amidst evolving climatic perturbations, setting the stage for targeted biotechnological strategies for conserving these relic taxa.

The synergism between plants and symbiotic organisms and innovative external treatments, epitomizes a paradigm shift in strategies to fortify plant robustness against environmental perturbations. Han et al. provided a comprehensive treatise on the symbiotic affiliations between plants and arbuscular mycorrhizal (AM) fungi, elucidating their role in promoting zinc (Zn) homeostasis in Eucalyptus grandis. Through comparative analyses between mycorrhized and non-mycorrhized specimens under differential Zn exposures, they observed enhanced vegetative metrics in symbiotically associated plants under elevated Zn regimes. In conjunction, the AM fungal consortium facilitated the absorption kinetics of essential ionome nutrients, with a concomitant genetic upregulation underpinning the beneficial role of this symbiotic association in Zn stress mitigation. In a subsequent investigative endeavor, Han et al. probed innovative methodologies to enhance cryogenic tolerance in *Vitis vinifera* L., particularly emphasizing the efficacy of Biodegradable Liquid Film (BLF). Their empirical analyses displayed a significant reduction in plant morbidity metrics in BLF-treated specimens. Concurrent biochemical profiling attributed this heightened cryotolerance to the amplification of specific osmoprotective agents and a diminution of oxidative perturbations. Furthermore, a discernible genetic modulation in response to cryogenic stress post-BLF application suggested its potentiality as a protective adjunct in horticultural practices.

In the face of growing environmental challenges, biotechnological advances promise enhanced resilience in vital crops. Leveraging genetics, molecular insights, and symbiotic partnerships amplifies the possibility of creating hardier crops. The data presented in this sub-theme highlights critical avenues for future research endeavors.

## Conclusion

These 30 articles collectively shed light on the intricate web of interactions within plants confronting various abiotic stress, offering new horizons for agriculture and deepening our understanding of plant stress adaptation mechanisms. This Research Topic provides a comprehensive overview of plants’ multifaceted landscape of abiotic stress adaptation. It unravels the intricate mechanisms governing plant responses to stressors, spanning physiological, biochemical, molecular, and biotechnological dimensions. This compilation underscores the importance of continued research and collaboration in plant science to address the pressing challenges posed by abiotic stressors in agriculture. We can bolster crop yields and food security by harnessing the insights gleaned from these studies and contribute to sustainable agriculture practices in a changing climate. Further exploration in this field holds the potential to shape the future of plant stress adaptation and pave the way for innovative solutions in the agriculture sector.

## Author contributions

MM: Writing – review & editing, Conceptualization, Data curation, Resources, Validation, Visualization, Writing – original draft. MM: Conceptualization, Project administration, Resources, Supervision, Writing – review & editing. MR: Project administration, Resources, Supervision, Writing – review & editing, Conceptualization. DM: Conceptualization, Project administration, Resources, Supervision, Writing – review & editing.

